# NAD(P)H:quinone oxidoreductase 1 determines radiosensitivity of triple negative breast cancer cells and is controlled by long non-coding RNA NEAT1

**DOI:** 10.7150/ijms.45706

**Published:** 2020-08-19

**Authors:** Li-Ching Lin, Hsueh-Te Lee, Peng-Ju Chien, Yu-Hao Huang, Mu-Ya Chang, Yueh-Chun Lee, Wen-Wei Chang

**Affiliations:** 1 Department of Radiation Oncology, Chi-Mei Foundation Medical Center, Tainan, Taiwan.; 2 School of Medicine, Taipei Medical University, Taipei, Taiwan.; 3 Chung Hwa University of Medical Technology, Tainan, Taiwan.; 4 Institute of Anatomy and Cell Biology, School of Medicine, National Yang Ming University, Taipei City, Taiwan.; 5 School of Biomedical Sciences, Chung Shan Medical University, Taichung, Taiwan.; 6 Department of Radiation Oncology, Chung Shan Medical University Hospital, Taichung, Taiwan.; 7 School of Medicine, Chung Shan Medical University, Taichung, Taiwan.; 8 Department of Medical Research, Chung Shan Medical University Hospital, Taichung 40201, Taiwan.

**Keywords:** NAD(P)H:quinone oxidoreductase 1, radiosensitivity, triple negative breast cancer, long non-coding RNA NEAT1

## Abstract

Radioresistant cells cause recurrence in patients with breast cancer after they undergo radiation therapy. The molecular mechanisms by which cancer cells obtain radioresistance should be understood to develop radiation-sensitizing agents. Results showed that the protein expression and activity of NAD(P)H:quinone oxidoreductase 1 (NQO1) were upregulated in radioresistant MDA-MB-231 triple-negative breast cancer (TNBC) cells. NQO1 knockdown inhibited the proliferation of NQO1 expressing Hs578t TNBC cells or the radioresistant MDA-MB-231 cells, whereas NOQ1 overexpression increased the survival of MDA-MB-231 cells, which lack of NQO1 expression originally, under irradiation. The cytotoxicity of β-lapachone, an NQO1-dependent bioactivatable compound, was greater in radioresistant MDA-MB-231 cells than in parental cells. β-lapachone displayed a radiosensitization effect on Hs578t or radioresistant MBDA-MB-231 cells. The expression of the long noncoding RNA NEAT1 positively regulated the NQO1 expression in radioresistant MDA-MB-231 cells at a translational level rather than at a transcription level. The inhibition of the NEAT1 expression through the CRISPR-Cas9 method increased the sensitivity of radioresistant MDA-MB-231 cells to radiation and decreased their proliferation, the activity of cancer stem cells, and the expression of stemness genes, including BMI1, Oct4, and Sox2. In conclusion, the NQO1 expression in triple-negative breast cancer cells determined their radiosensitivity and was controlled by NEAT1. In addition, NOQ1 bioactivatable compounds displayed potential for application in the development of radiation sensitizers in breast cancer.

## Introduction

Radiotherapy is clinically used in breast cancer to prevent local recurrence after patients undergo breast-conserving surgery, but the efficiency of radiotherapy among breast cancer subtypes varies. Clinical data have revealed that triple-negative breast cancer (TNBC) and breast cancers with a high expression level of human epidermal growth factor receptor 2 (HER2) have a higher local recurrence risk than other subtypes 1. TNBC is a type of breast cancer that does not express hormone receptors (estrogen and progesterone receptors) and HER2, and it accounts for 15%-20% of the total incidence of breast cancer 2. TNBC is an aggressive subtype of breast cancer with a high proliferation rate and a short median time to relapse and death 3. Kyndi et al. 4 reported that the reduction of the local recurrence rate of TNBC after patients are subjected to a modified radical mastectomy is less than that of luminal-type breast cancers; this result suggests the radioresistant characteristic of TNBC cells. As such, understanding the molecular mechanisms of the radioresistant phenotype of TNBC cells is beneficial to the development of radiation sensitizers.

The main mechanism of cancer radiotherapy involves the induction of DNA damage directly by ionizing radiation or indirectly by the generation of reactive oxygen species (ROS) 5. Antioxidant molecules, such as antioxidant enzymes, including superoxide dismutase or glutathione reductase, with increased expression levels within cancer cells are responsible for the radioresistance of cancer 6. Oxidative stress induces NAD(P)H:quinone oxidoreductase 1 (NQO1), which is widely expressed in cells and functions as a two-electron reductase to detoxify quinones and related substrates or to maintain intracellular antioxidants 7. NQO1 is increased in many types of cancers, including breast cancer. The high NQO1 expression in breast cancer is correlated with the late clinical stage and the poor overall survival rate 8. Treatments involving dicumarol, an NQO1 inhibitor, potentiate the cytotoxic effects induced by chemotherapy drugs, such as cisplatin 9 or gemcitabine 10, on cancer cells; this result implies the involvement of NQO1 in cancer chemoresistance. Park et al. 11 found that treatment with β-lapachone, an NQO1-bioactivatable compound, can sensitize NQO1-expressing MDA-MB-231 cells to ionizing radiation. However, the role of NOQ1 in cancer radioresistance remains unclear.

Long noncoding RNAs (LncRNAs) are >200 bp polyadenylated ncRNAs transcribed by RNA polymerase II 12. LncRNAs play roles in the epigenetic control of gene expression, cell cycle control, DNA damage, microRNA silencing, and signal transduction pathways 13. Several lncRNAs also participate in carcinogenesis. In breast cancer, the knockdown of metastasis-associated lung adenocarcinoma transcript 1 in a mouse model of breast cancer results in the slow growth of tumors and the loss of their metastatic ability 14. The expression of nuclear paraspeckle assembly transcript 1 (NEAT1) is required for the survival of breast cancer cells 15, and a high NEAT1 expression is correlated with the poor survival of patients with breast cancer 16. Hypoxia-induced NEAT1 expression accelerates the proliferation and inhibits the apoptosis of breast cancer cells 16. Hypoxia-induced NEAT1 expression accelerates the proliferation of breast cancer cells and inhibits their apoptosis 17. In cervical cancer cells, NEAT1 enhances radioresistance by inhibiting miR-193b-3p and upregulating cyclin D1 18. However, the involvement of NEAT1 in the NOQ1 expression and the radioresistance of TNBC cells remain unclear.

## Materials and Methods

### Chemicals

β-Lapachone (Cayman Chemical, Ann Arbor, MI) was dissolved in dimethyl sulfoxide (DMSO; Sigma-Aldrich, St. Louis, MO, USA) as a stock of 10 mg/ml and stored at -20 °C. SP600125 (Sigma-Aldrich) was dissolved in DMSO as a stock of 10 mM and stored at -20 ºC.

### Cell culture

MDA-MB-231 or Hs578t cells were (Bioresource Collection and Research Center in the Food Industry Research and Development Institute, Hsinchu City, Taiwan) maintained at 37 ºC in a 5% CO_2_ incubator with DMEM/F12 medium containing 10% fetal bovine serum (Biological Industries, Kibbutz Beit-Haemek, Israel) and 1X penicillin/streptomycin (Biological Industries).

### Establishment of radioresistant MDA-MB-231 cells

Radioresistant MDA-MB-231 cells (231-RR cells) were established by repeatedly exposing them to 2 Gy irradiation 19. Briefly, 5×10^5^ cells were seeded into a 6 cm cell culture dish and allowed attachment at 37 ºC overnight. The dish with the attached cells was then exposed to 2 Gy irradiation by using an Elekta AxesseTM linear accelerator (Elekta AB, Stockholm, Sweden) at a dose rate of 6 Gy min^-1^. After irradiation, a fresh culture medium was used for expansion to 80% confluence, and the cells were cultured in an incubator with 5% CO_2_ at 37 ºC. The expanded cells were then harvested for the next round of 2 Gy irradiation until a total dose of 40 Gy was administered.

### Clonogenic assay

The cells were seeded into a six-well plate with densities of 500 cells/well for MDA-MB-231 cells and 1,000 cells/well for Hs578t cells and allowed attachment at 37 ºC overnight. The plates with the attached cells were then exposed to different irradiation doses by using the Elekta AxesseTM linear accelerator as indicated above. After irradiation, the cell culture media were changed to fresh ones, and the cells were cultured in an incubator with 5% CO_2_ at 37 ºC for 14 days. The formed colonies were fixed with 3.7% formaldehyde/PBS at room temperature for 5 min and then stained with crystal violet. The seeding number of the NQO1-knocked down cells was increased to 2,000 cells/well for 231-RR cells and 5,000 cells/well for Hs578t cells to determine the radiosensitivity of the NQO1-knocked down cells. The survival fractions after irradiation was calculated in accordance with the report of Franken et al. 20 and drawn with GraphPad Prism (version 5.0, GraphPad Software, San Diego, CA, USA) by using a linear quadratic model with a formula of:


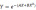
(1)

where *X* is the radiation dose, and *Y* is the survival fraction. The estimated survival fraction of 0.5 and the sensitizer enhancement ratio of 50% inhibition (SER50) were calculated using previously reported formulas 21.

### Western blot analysis

Total cellular proteins were collected through the lysis of harvested cells with RIPA buffer (GeneTex Inc., Hsinchu City, Taiwan). Afterward, 25 μg of proteins was separated via SDS-PAGE and transferred onto PVDF membranes (Immobilon-P, Merckmillipore, Danvers, MA, USA). The membranes were blocked with 5% skimmed milk, incubated with primary antibodies at 4 ºC overnight, and incubated with horseradish peroxidase (HRP)-conjugated specific secondary antibodies at room temperature for 1 h. The signals were developed by incubating with an enhanced chemiluminescence substrate (PerkinElmer, Waltham, MA, USA) and captured with a luminescent image analyzer (Fusion Solo, Vilber Lourmat Deutschland GmbH, Germany). The following antibodies were used in this study: mouse monoclonal IgG anti-Myc tag antibody (Proteintech Group Inc., Rosemont, IL, USA); mouse monoclonal IgG anti-NQO1 (Cat. No. sc-32793), anti-Nrf2 (Cat. No. sc-365949), and anti-JNK (Cat. No. sc-7345) antibodies (Santa Cruz Biotechnologies Inc., Dallas, TX, USA); anti-phosphorylated JNK antibody (Cat. No. 4668S; Cell Signaling Technology, Danvers, MA, USA); rabbit polyclonal IgG anti-GAPDH antibody (Cat. No. GTX100118; GeneTex Inc., Hsinchu City, Taiwan); and mouse monoclonal IgG anti-β-actin (Cat. No. A5441; Sigma-Aldrich, St. Louis, MO, USA).

### NQO1 activity assay

An NQO1 activity assay kit (Cat. No. ab184867; Abcam Plc., Cambridge, UK) was based on the reduction of menadione with an NADH cofactor and the simultaneous reduction of WST1 to form WST1-formazan, which could be read on the basis of the absorbance at 440 nm wavelength. Dicumarol was used as the NQO1 inhibitor, and the NQO1 activity was calculated by subtracting OD with dicumarol from OD without dicumarol.

### Manipulation of NQO1 expression

For the NQO1 overexpression, the NQO1 expression vector (Cat. No. HG12046-CM; Sino Biological Inc., Beijing, China) was mixed with HyFect^TM^ DNA Transfection Reagent (Leadgene Biomedical Inc., Tainan, Taiwan) at a ratio of 1 μg of DNA:3 μl of reagent in 50 μl of Opti-MEM^TM^ medium (Thermo Fisher Scientific Inc., Waltham, MA, USA) at room temperature for 15 min. The DNA/reagent complexes were then added to MDA-MB-231 cells and incubated at 37 °C overnight. Afterward, a fresh medium containing 400 μg/ml hygromycin B (Roche Diagnostics GmbH, Mannheim, Germany) was prepared for selection for 96 h. The surviving cells were then used for further experiments. For the NQO1 knockdown, the cells were transduced with NQO1-specific shRNA (Clone No. TRCN0000350362) or LacZ-specific shRNA (Clone No. TRCN0000231722) carrying a lentivirus (the National RNAi Core Facility at Academia Sinica, Taipei, Taiwan) with 8 μg/ml polybrene (Sigma-Aldrich) at 37 ºC overnight. A fresh medium containing 2 μg/ml puromycin (TOKU-E, Bellingham, WA, USA) was also prepared for selection for 48 h. The surviving cells were harvested and used for further experiments.

### Inhibition of NEAT1 by CRISPR-Cas9

CRISPR-Cas9-mediated gene inhibition was carried out by transfecting a nonviral NEAT1 sgRNA all-in-one vector (Cat. No. EF177379; Applied Biological Materials Inc., Richmond, BC, Canada) by using a HyFect^TM^ DNA transfection reagent in accordance with the protocol described above. After transfection, the cells were selected with 2 μg/ml puromycin for 96 h and harvested for the selection of single colonies. The cells derived from different single colonies were used for examining the NEAT1 expression via quantitative RT-PCR as described below, and the cells with a decreased NEAT1 expression were utilized for further experiments.

### Quantitative RT-PCR

Total cellular RNA was extracted using an RNA extraction kit (Zymo Research, Irvine, CA, USA), and 1 μg of the extracted total RNA was used for cDNA synthesis with a first-strand cDNA synthesis kit with random hexamer primers (Fermentas Inc., Waltham, MD, USA). Target genes were quantified and detected with SYBR Green-based quantitative PCR method by using a PCRmax Eco 48 real-time PCR system (PCR max, Staffordshire, UK) and the PCR cycle condition was setup according to our previous report 19. The primer sets using in this study were listed as follows:

- NQO1-F: 5'-AAAGGACCCTTCCGGAGTAA-3';
- NOQ1-R: 5'-CCATCCTTCCAGGATTTGAA-3';
- NEAT1-F: 5'-TAGTTGTGGGGGAGGAAGTG-3';
- NEAT1-R: 5'-ACCCTGCGGATATTTTCCAT-3';
- BMI1-F: 5'-AATCCCCACCTGATGTGTGT-3';
- BMI1-R: 5'-GCTGGTCTCCAGGTAACGAA-3';
- Oct4-F: 5'- GGTCCGAGTGTGGTTCTGTA-3';
- Oct4-R: 5'- CGAGGAGTACAGTGCAGTGA-3';
- Sox2-F: 5'- AACCCCAAGATGCACAACTC-3';
- Sox2-R: 5'- CGGGGCCGGTATTTATAATC-3'.

### Tumorsphere formation assay

For primary tumorsphere cultivation, the cells were suspended as 1×10^3^ cells/2 ml in DMEM/F12 containing 0.4% BSA, 20 ng/ml EGF, 20 ng/ml bFGF, 0.5X B27 supplement, 4 μg/ml heparin, 5 μg/ml insulin, and 1 μM hydrocortisone and seeded into each well of suspension culture in six-well plates (Greiner Bio-One International GmbH, Kremsmünster, Austria). Then, 0.5 ml of fresh media was added to the wells on day 3, and the number of formed tumorspheres was counted on day 7. The counting criterion of tumorspheres was set as the diameter larger than 50 μm. Afterward, the formed primary tumorspheres were collected using a 70 μm cell strainer (BD Biosciences, San Jose, CA, USA) and dissociated into single cell suspension with HyQTase solution (Hyclone Laboratories Inc., South Logan, UT, USA). Then, secondary tumorspheres were cultivated in the same manner as primary tumorsphere cultivation, but the initial number of seeding cells was 500 cells/well.

## Results

### NQO1 expression determines the radiosensitivity of TNBC cells

**Figure 1:**
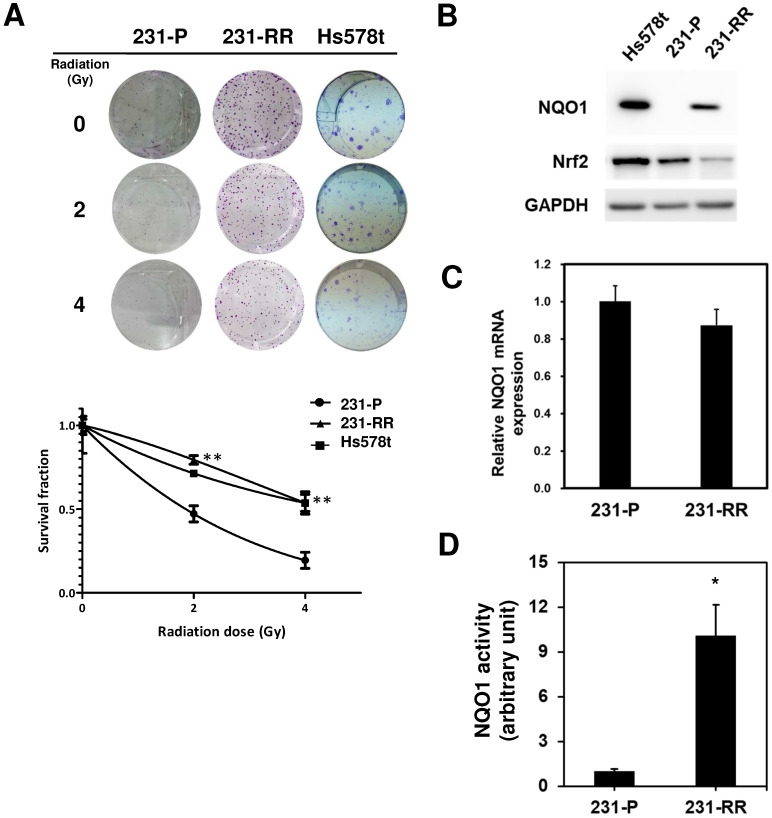
** The protein expression and activity of NQO1 is upregulated in radioresistant MDA-MB-231 cells.** (**A**) Radiosensitivity of Hs578t, MDA-MB-231 (231-P) or the radioresistant MDA-MB-231 (231-RR) cells was determined by clonogenic assay. Data were presented as survival fractions which were calculated by the linear-quadratic model using GraphPrism software. **, *p<* 0.01; *, *p<* 0.05. (**B**) NQO1 protein expressions in Hs578t, 231-P, or 231-RR cells were determined by western blot. GAPDH was used as a loading control. (**C**) The NQO1 mRNA expression in 231-P or 231-RR cells was determined by qRT-PCR method and data were presented as relative expression level when compared to 231-P cells. (**D**) NQO1 activity in cellular protein preparation was determined as the described in Materials and Methods section. *, *p<* 0.05.

**Figure 2:**
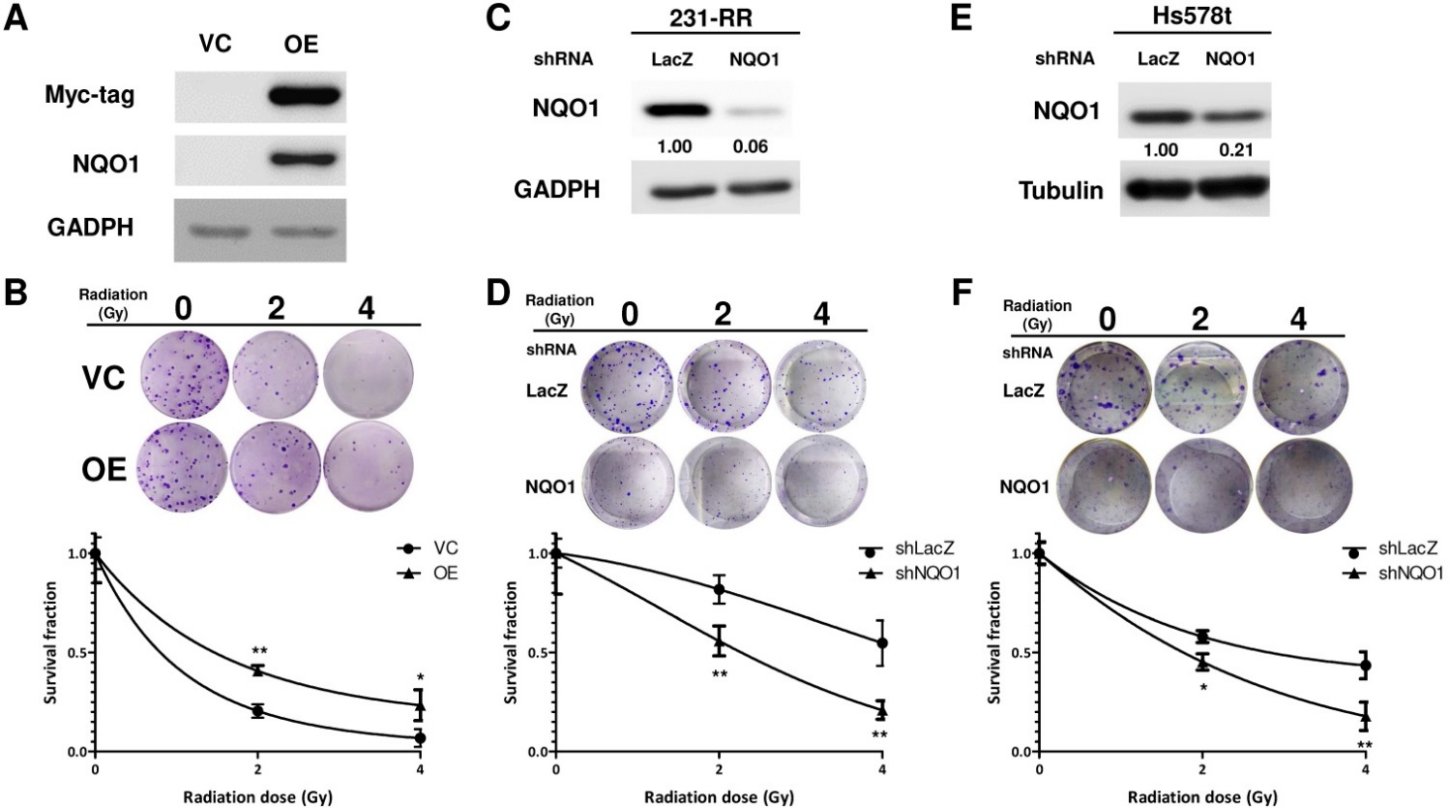
** NQO1 expression level influences radiosensitivity of MDA-MB-231 cells.** (A, B) Overexpression of NOQ1 was performed in MDA-MB-231 cells by transfection of myc-tagged NQO1 expression vector and confirmed by western blot (**A**). VC, vector control; OE, overexpression cells. VC or OE cells were then seeded into 6-well-plate and performed irradiation as indicated dosage followed by clonogenic assay (**B**). The colonies were visualized and counted after crystal violet staining. Data were presented as survival fractions which were calculated by the linear-quadratic model. *, *p<* 0.05; **, *p<* 0.01. (**C-F**) Knockdown of NQO1 in radioresistant MDA-MB-231 cells (231-RR, C and D) or Hs578t (E and F) was performed by transduction of a NQO1-specific shRNA carrying lentivirus. The LacZ-specific shRNA clone was used as a negative control. After puromycin selection, the viable cells were harvested and checked NQO1 expression by western blot (C and E). The radiosensitivity of cells were determined by clonogenic assay and the data were presented as survival fraction which was calculated by the linear-quadratic model (D and F). *, *p<* 0.05; **, *p<* 0.01.

The radiosensitivity of Hs578t, MDA-MB-231, and the established radioresistant cells from the MDA-MB-231 cells (named as 231-RR cells) was first examined by repeatedly exposing them to 2 or 4 Gy radiation to investigate the molecular mechanisms underlying the radioresistance of TNBC cells. A clonogenic assay was performed to examine the survival fractions after different irradiation doses were administered. The results revealed that the survival rate of Hs578t or 231-RR cells was significantly greater than that of MDA-MB-231 cells after they were exposed to 2 or 4Gy irradiation (Figure 1A). The estimated radiation doses for the survival fraction of 0.5 for MDA-MB-231, Hs578t, or 231-RR were 1.70, 4.43, or 4.56 Gy, respectively. Park et al. 22 previously demonstrated that β-lapachone, a NQO1-dependent bioactivatable compound, can enhance the cytotoxicity of ionizing radiation in NQO1-overexpressing MDA-MB-231 cells 11; the results from Park et al. suggest that NQO1 expression may influence the radiosensitivity of TNBC cells. The protein expression of NQO1 among Hs578t, 231-RR cells and parental MDA-MB-231 cells was then examined through Western blot analysis. The results showed that the NQO1 protein expression was observed in Hs578t and 231-RR cells but not in MDA-MB-231 cells (Figure 1B). However, the mRNA level of NQO1 did not differ between 231-RR and MDA-MB-231 cells (Figure 1C). In addition to protein expression, the activity of NQO1 was higher in 231-RR cells than in MDA-MB-231 cells (Figure 1D). These data suggest that the NQO1 protein level is negatively correlated with radiosensitivity. To investigate the involvement of NQO1 in the radiosensitivity of TNBC cells, NQO1 was ectopically overexpressed in MDA-MB-231 cells by transient transfection of a NQO1 expressing vector, and the cell survival after irradiation was examined. The overexpression of NQO1 was confirmed through Western blot (Figure 2A). The survival fraction of NQO1-overexpressing MDA-MB-231 cells was greater than that of the vector control ones after irradiation at 2 or 4 Gy (Figure 2B). The sensitizer enhancement ratio of the fractional survival of 50% inhibition (SER50) was further calculated as previously described by Naumann et al. 21 to evaluate the changes in radiosensitivity after NQO1 overexpression. The results revealed that SER50 decreased to 0.546 in the NQO1-overexpressing MDA-MB-231 cells compared to controls (Table 1). In order to further confirm the importance of NQO1 to the radiation response of TNBC cells, RNA interference was applied to knock down the NQO1 expression in 231-RR or Hs578t cells. The protein expression of NQO1 was obviously inhibited in 231-RR (Figure 2C) or Hs578t (Figure 2E) cells after the lentiviral delivery of NQO1-specific shRNAs. Clonogenic assay results demonstrated that the knockdown of NQO1 in 231-RR (Figure 2D) or Hs578t (Figure 2F) cells significantly reduced the survival fractions at 2 or 4 Gy irradiation. Furthermore, SER50 of NQO1-knocked down 231-RR or Hs578t cells increased to 1.91 or 1.59, respectively (Table 1). These data indicated that the NQO1 expression level contribute to determine the radiosensitivity of TNBC cells and suggested that NQO1 bioactivatable agents could be used as alternative therapeutics in suppressing the radioresistance of TNBC cells.

### β-Lapachone increases the radiation sensitivity of radioresistant TNBC cells

**Figure 3:**
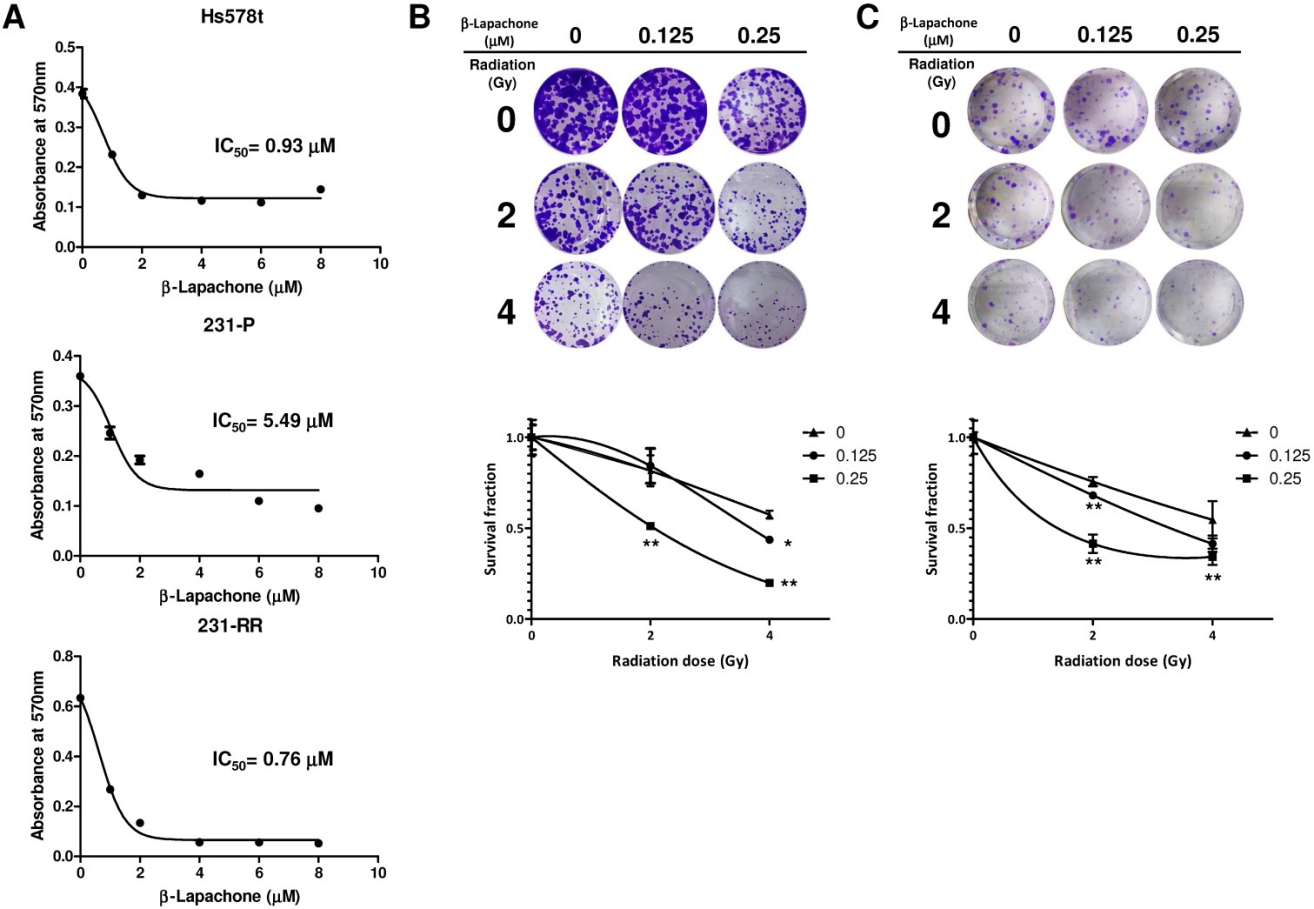
** β-lapachone sensitizes radioresistant MDA-MB-231 to radiation treatment.** (**A**) Hs578t, MDA-MB-231 (231-P) or radioresistant MDA-MB-231 (231-RR) cells were treated with the indicated concentration of β-lapachone in wells of 96-well-plate for 72 hours. Cell viability was determined by addition of MTT reagent and read the absorbance at 570 nm after dissolved by DMSO. IC_50_ values were calculated with a web-based IC50 calculator (https://www.aatbio.com/tools/ic50-calculator). (**B, C**) 231-RR (B) or Hs578t (C) cells were seeded into 12-well-plates as 200 cells/well (231-RR) or 1×10^3^ cells/well (Hs578t) in presence of indicated concentration of β-lapachone followed by radiation treatment (2 or 4 Gy). The survival fractions were then determined by clonogenic assay and calculated by the linear-quadratic model. *, *p<* 0.05; **, *p<* 0.01.

Considering the high expression level of NQO1 in 231-RR or Hs578t cells, we next examined whether the 231-RR or Hs578t cells are more sensitive to the NQO1-dependent bioactivatable compound β-lapachone than the NQO1 unexpressed MDA-MB-231 cells. Using the MTT assay, we found that the half maximal inhibitory concentrations (IC_50_) of β-lapachone for MDA-MB-231, 231-RR, and Hs578t cells were 5.49, 0.76, and 0.93 μM, respectively (Figure 3A). These results also confirmed the increased NQO1 activity in the radioresistant 231-RR cells. We next examined the effect of β-lapachone treatment on the radiosensitivity of 231-RR and Hs578t cells. The β-lapachone treatment of the radioresistant 231-RR cells (Figure 3B) or Hs578t cells (Figure 3C) significantly reduced the colony number under 2 or 4 Gy irradiation. SER50 increased in the β-lapachone-treated cells (Table 1) in a dose-dependent manner. These results clearly indicate that the increased NQO1 activity could be used as a sensitization strategy to enhance radiosensitivity in TNBC cells through NQO1-dependent bioactivatable compounds, such as β-lapachone.

### LncRNA NEAT1 positively regulates NQO1 expression and cancer stem cell activity in TNBC cells

**Figure 4:**
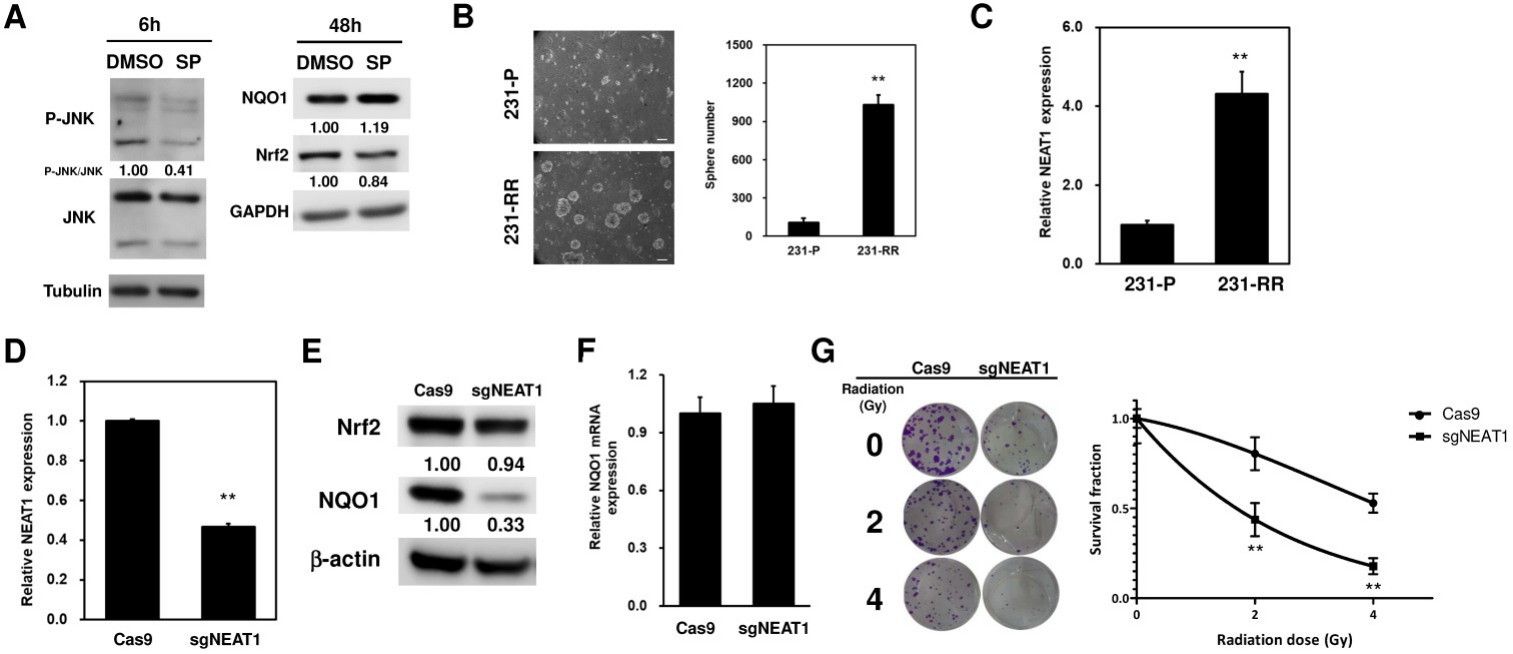
** LncRNA NEAT1 positively regulates NOQ1 expression in MDA-MB-231 cells.** (**A**) SP600125 (SP), the JNK inhibitor, was used as 10 μM and the JNK activation was determined by western blot analysis of p-JNK at 6h (left panel). The expression of NQO1 or Nrf2 in radioresistant MDA-MB-231 cells after SP treatment was examined by western blot analysis (right panel). α-tubulin or GAPDH was used as the loading control. (**B**) The CSC activity of MDA-MB-231 (231-P) or radioresistant MDA-MB-231 (231-RR) cells was determined by tumorsphere cultivation. The numbers of formed tumorspheres were pictured (left panel) and counted (right panel) at Day 7 post-seeding. **, *p<* 0.01. (**C**) NEAT1 expression in 231-P or 231-R cells was determined by SYBR Green based quantitative RT-PCR. **, *p<* 0.01. (**D to G**) Inhibition of NEAT1 expression in 231-RR cells was performed by transfection of non-viral vector containing Cas9 and NEAT1 sgRNA sequence (sgNEAT1) and selected by puromycin. A Cas9-expressing plasmid was used as a negative control (Cas9). NEAT1 expression after puromycin selection was determined by qRT-PCR (D). NQO1 expression was detected by western blot (E). The inserted numbers indicated relative expression level after compared to Cas9 group. The NQO1 mRNA expression of Cas9- or sgNEAT1-transfected cells was determined by qRT-PCR (F). Cas9- or sgNEAT-transfected 231-RR cells were irradiated for 2 or 4 Gy and the fractional survivals of cells were determined by clonogenic assay and calculated by the linear-quadratic model (G). **, *p<* 0.01.

**Figure 5:**
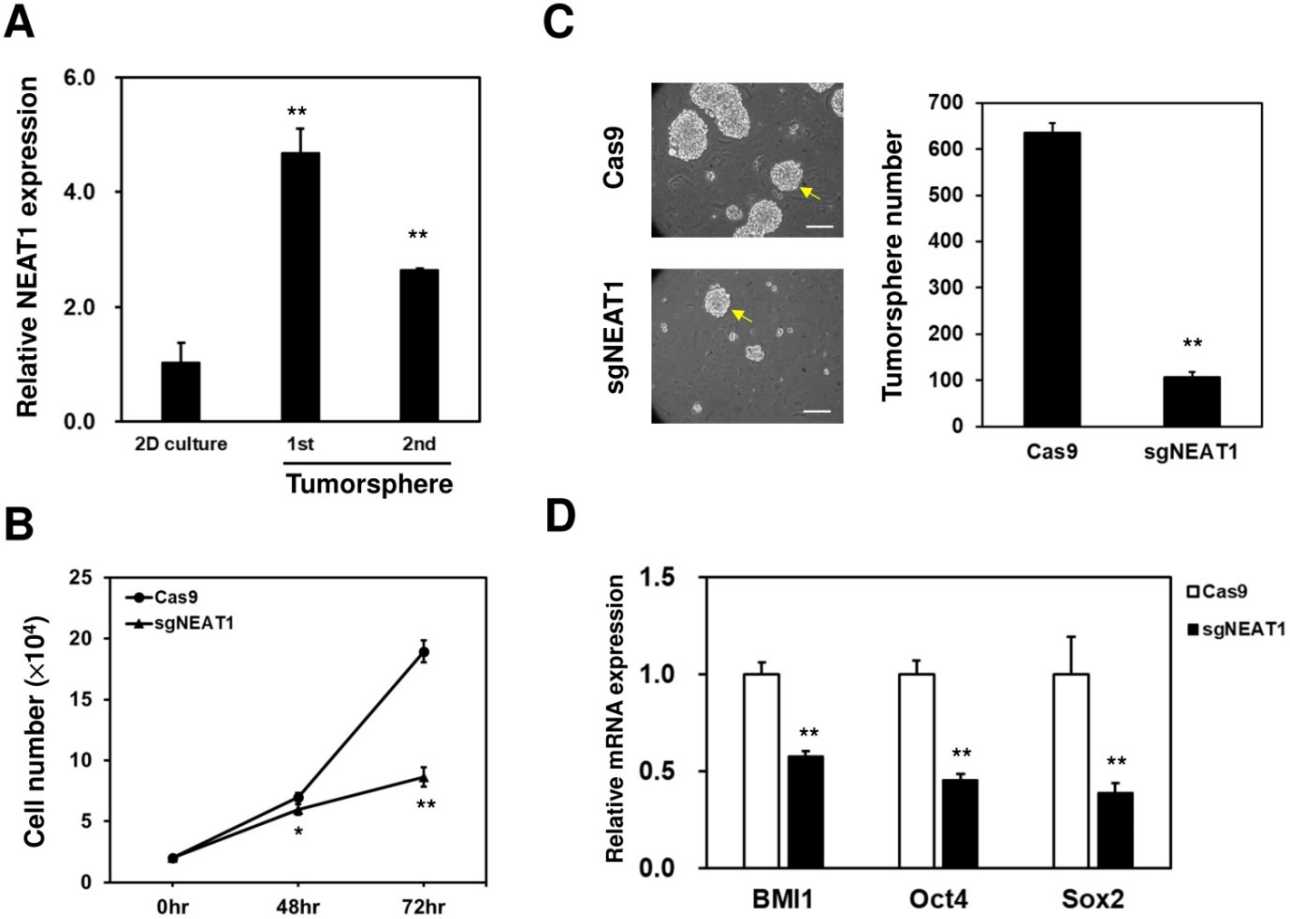
** NEAT1 expression is required for CSC maintenance in radioresistant MDA-MB-231 cells.** (**A**) NEAT1 expression among 2-dimension (2D) culture cells, primary (1^st^) or secondary (2^nd^) tumorspheres of radioresistant MDA-MB-231 cells was determined by qRT-PCR. **, *p<* 0.0.1 when compared to 2D culture group. (**B**) Cells were seeded in a 96-well-plate as 1000 cells/well and cultured for 24, 48, and 72 hours. The cell growth curves were by MTT reagent. *, *p<*0.05; **, *p<* 0.01. (**C**) Inhibition of NEAT1 in radioresistant MDA-MB-231 cells was performed as described in Figure 4C. Self-renewal capability of the viable cells after transfection of Cas9 or Cas9/sgNEAT1 non0viral plasmid was determined by tumorsphere cultivation. **, *p<* 0.01. The insert bars indicated 50 μm in length. Arrows indicated the reference size of tumorsphere for counting. (**D**) The mRNA expression of BMI1, Oct4, or Sox2 in radioresistant MDA-MB-231 cells after NEAT1 inhibition was determined by qRT-PCR method. **, *p<* 0.01.

Literature data report that NQO1 expression could be positively regulated through the c-Jun N-terminal kinase (JNK)-induced activation of nuclear factor erythroid-2-related factor 2 (Nrf2) 23. Therefore, to investigate whether this could be the molecular mechanism underlying the upregulation of NQO1 in 231-RR cells, we treated the 231-RR cells with the JNK inhibitor SP600125 and examined the NQO1 expression. We found that the p-JNK expression was reduced 6 h after SP600125 treatment (Figure 4A). This result demonstrated that JNK inhibition was successfully obtained. However, the expression level of NQO1 did not reduce at 48 h after the treatment of SP600125 (Figure 4A). These results suggested that the upregulation of NQO1 in 231-RR cells was not mediated by JNK activation. The radioresistance of cancers can be achieved through the existence of cancer stem cells (CSCs) 24. We next hypothesized that the radioresistance feature of 231-RR cells could be mediated through the increased CSC activity. Using tumorsphere cultivation as an indicator for the examination of the CSC activity 25, we found an increased number of tumorspheres in the 231-RR cells in comparison with parental MDA-MB-231 cells (Figure 4B), indicating that the CSC population increased in 231-RR cells. Considering the involvement of lncRNA NEAT1 in the maintenance of CD24^-^CD44^+^ CSC population in breast cancer cells 17 and in the enhancement of the radioresistance of cervical cancer cells 18, we next hypothesized that the expression of NEAT1 could be upregulated in radioresistant 231-RR cells, possibly leading to the increased CSC activity followed by redioresistance. Utilizing the qRT-PCR method to detect the expression of NEAT1 between the radioresistant and parental MDA-MB-231 cells, we observed that NEAT1 was upregulated in the radioresistant 231-RR cells (Figure 4C). We further hypothesized that NEAT1 positively regulated NQO1 expression. We also applied the CRISPR-Cas9 method to inhibit NEAT1 expression in the MDA-MB-231 cells (Figure 4D). The results showed that NQO1 protein expression obviously decreased in the sgNEAT1-transfected 231-RR cells (Figure 4E) without influencing the protein expression of Nrf2, the well-known transcription factor for NQO1 expression 26, and the mRNA expression of NQO1 (Figure 4F). This suggests that the upregulation of NEAT1 expression involves in the NQO1 induction in radioresistant TNBC cells through a Nrf2 independent mechanism. The survival fractions after radiation were found to be significantly reduced in the NEAT1-inhibited 231-RR cells (Figure 4G), suggesting that the expression of NEAT1 promoted the radioresistance of TNBC cells. To further investigate the importance of NEAT1 in the maintenance of CSCs in MDA-MB-231 cells, we used the cultivated tumorsphere to enrich CSC population and examined the NEAT1 expression via qRT-PCR. The results revealed that the increased NEAT1 was found in the enriched CSCs as primary or secondary tumorspheres from the MDA-MB-231 cells (Figure 5A). After inhibiting NEAT1 expression by CRISPR-Cas9 method, we firstly observed the reduced cell growth at 72 h (Figure 5B). We then collected the viable Cas9- or sgNEAT1-transfected cells to examine the CSC activity via tumorsphere cultivation and found that the number of tumorspheres significantly decreased in NEAT1-inhibited MDA-MB-231 cells (Figure 5C). We also observed the decreased expression of several cancer stemness genes, including BMI1, Oct4, and Sox2 (Figure 5D), which maintain breast CSCs 27-29. These data indicated that NEAT1 expression positively regulated the proliferation of MDA-MB-231 cells and the maintenance of the CSC population within MDA-MB-231 cells. These results also suggested that the involvement of lncRNA NEAT1 in the radioresistance of TNBC cells could be mediated through its positive regulatory role in the maintenance of CSCs.

## Discussion

The increased NQO1 expression in cancer is linked to an increased Nrf2 expression 30. However, we did not find a difference in the Nrf2 expression between 231-P and the radioresistant 231-RR cells (Figure 4A). We did not also observe a significant difference in the mRNA expression of NQO1 between 231-P and 231-RR cells (Figure 1C). This result suggested that the induction of NQO1 by radiation was independent of Nrf2-mediated transcriptional regulation. Recently, Beinse et al. 31 conducted immunohistochemistry analysis and found no correlation between Nrf2 and NQO1 expression levels in endometrial cancer tissues. Taken together, these results indicated that an Nrf2-independent regulation of NQO1 expression possibly existed in cancers.

NQO1 upregulation in cancers leads to the development of diagnostic or therapeutic agents based on NQO1 activity. The generation of hydroquinone by NQO1 reduction from β-lapachone can produce superoxides that lead to cell death 32. Park et al. 11 demonstrated the radiosensitizing effect of β-lapachone on MDA-MB-231 cells after the forced expression of NQO1. The combination of β-lapachone with ionizing radiation in NQO1-overexpressing MDA-MB-231 cells causes ROS generation, followed by JNK activation, through treatment-induced endoplasmic reticulum stress to trigger mitochondrial apoptotic cell death 11. Our data also clearly demonstrated that β-lapachone treatment sensitized the radioresistant 231-RR cells (Figure 3B) or Hs578t cells (Figure 3C) toward radiation, suggesting that β-lapachone could be potentially developed as a radiosensitizer in breast cancers with an increased NQO1 activity. Given the ubiquitous expression feature of NQO1 33, cancer targeting must be achieved to avoid side effects. Huang et al. 34 previously reported a cancer-targeting NQO1 prodrug with an indolequinone structure linked to an integrin αvβ3-targeting peptide c (RGDyK). Recently, Hill et al. 35 reported that αvβ3 is generally expressed in TNBC tissues. The potential of TNBC-targeting peptides conjugated to NQO1 bioactivatable compounds as radiosensitizers may be worthy of clinical evaluation.

NEAT1 regulates microRNA expression. In breast cancer cells, NEAT1 is a competing endogenous RNA (ceRNA) that inhibits the expression/function of several miRNAs, such as miR-211-5p 36, miR-214 37, or miR-218-5p 38. In addition to ceRNA, NEAT1 induces DNA methylation in the promoter of miR-129-5p, leading to the downregulation of miR-129-5p 39. We suggested that NEAT1 might function as a ceRNA to sponge NQO1-targeting miRNAs in 231-RR cells because of the unchanged mRNA expression of NQO1 between 231-P and 231-RR cells (Figure 1C) or in the sgNEAT1-transfected 231-RR cells (Figure 4F). From the TargetScan database (http://www.targetscan.org/vert_72/), miR-129-5p, miR-211-5p, miR-214-5p, and miR-218-5p might bind to the 3ʹ-UTR of the mRNA of NQO1 (Supplementary information, Figure S1). This result suggested that the positive regulatory role of NEAT1 in NQO1 expression was potentially mediated by suppressing NQO1-targeting miRNAs because the mRNA level was not changed in the radioresistant MDA-MB-231 cells (Figure 1C) and in NEAT1-knocked out cells (Figure 4F). However, further investigation is needed to prove this hypothesis. LncRNAs have also been reported to regulate the stability of intracellular proteins. For example, lncRNA GUARDIN sustains the protein stability of breast cancer 1 for maintaining genome stability 40. NEAT1 also stabilizes PTEN-induced kinase 1 in SH-SY5Y neuroblastoma cells to promote autophagy 41. In colorectal cancer cells, NEAT1 directly binds to the DDX5 protein, maintains its stability, and activates the Wnt signaling pathway 42. In our data, the mRNA level of NQO1 was not changed in NEAT1-inhibiting 231-RR cells (Figure 4F), but the NQO1 protein was reduced (Figure 4E). This result suggested that NEAT might regulate the protein stability of NOQ1 in 231-RR cells, but this finding should be further investigated.

In addition to the NQO1-regulating role of NEAT1, the tumorsphere formation capability radioresistant MDA-MB-231 cells was suppressed after inhibition of NEAT1 (Figure 5C) suggesting that the self-renewal capability of the CSC population within MDA-MB-231 cells was positively regulated by NEAT1. These data were consistent with those reported by Shin et al. 17, who demonstrated that lncRNA NEAT1 in TNBC cells regulates the cell cycle progression and that the knockdown of NEAT1 in TNBC cells decreases CSC populations, including CD24-CD44+ or Sox2+ cells. We also found the proliferation of radioresistant MDA-MB-231 was suppressed (Figure 5B), which suggests that NEAT1 also involves in the cell growth of whole populations of cancer cells. The role of NEAT1 in cancer radioresistance may also involve other cellular proteins through the direct interaction or its ceRNA activity. Chen et al. found that NEAT1 could recruit EZH2 (enhancer of zeste 2), a histone methyltransferase within the Polycomb repression complex 2, to promote glioblastoma progression 43. The increased EZH2 expression in prostate cancer was to be associated with metastastic recurrence after radiotherapy 44. Han et al. previously demonstrated that NEAT1 could function as a ceRNA to inhibit miR-193b-3p in cervical cancer cells, which led to the upregulation of cyclin D1 to promote radioresistance 18. Taken together, our data and these previous reports suggested that targeting NEAT1 expression may be used as a basis for developing a novel therapeutic strategy for TNBCs. The function of lncRNAs can be inhibited by transfecting siRNA or antisense oligonucleotides 45. However, the inefficient delivery of nucleic acid drugs limits their clinical applications 46. Spherical nucleic acids (SNAs) are composed of densely and highly oriented nucleic acids covalently attached to the surface of a spherical nanoparticle core 47. Unlike linear nucleic acids, SNAs can be rapidly internalized by a broad range of mammalian cells 48. SNA gold nanoparticles composed of siRNA targeting the Bcl-2-like protein 12 sequences have been developed and used for early phase 1 clinical trial in patients with recurrent glioblastoma 49. Using SNAs to carry siRNAs can overcome the issues related to cellular or even tissue delivery in the human body 50. Furthermore, SNAs composed of NEAT1-targeting sequences can be used to examine their radiosensitizer potential or therapeutic effect on TNBCs.

## Conclusions

Our data demonstrated that the expression and activity of NQO1 were greatly upregulated in TNBCs with a radioresistant phenotype. NQO1 bioactivatable compounds, such as β-lapachone, displayed a radiosensitizer potential in TNBC cells. Furthermore, the protein expression of NQO1 was controlled by lncRNA NEAT1, which positively regulated the CSC activity in TNBC cells. The inhibition of the NEAT1 expression also sensitized TNBC cells to radiation treatment. Targeting NQO1 or lncRNA NEAT1 could be an effective strategy for the development of radiosensitzers in TNBC therapy.

## Supplementary Material

Supplementary figure S1.

## Figures and Tables

**Table 1:** Summary of the values of sensitizer enhancement ratio for an estimated 50% inhibition of fractional survival (SER50)^a^

Experiments		SER50
Radioresistant MDA-MB-231 cells^b^		0.38
NQO1 overexpression^c^		0.55
Knockdown of NQO1^d^		
231-RR		1.91
Hs578t		1.59
β-lapachone (μM)^e^		
231-RR	0.125	1.26
	0.25	2.24
Hs578t	0.125	1.36
	0.25	3.18
sgNEAT1^f^		2.50

a: SER50 was calculated according to the formula as the report from Naumann et al.^21^; b: compared to parental MDA-MB-231 cells; c: compared to vector control cells; d: compared to sh-LacZ transduced cells; e: compared to 0.1% DMSO control cells; f: compared to Cas9 transfected cells.
