# Supplementary material for: NAD(P)H:quinone oxidoreductase 1 determines radiosensitivity of triple negative breast cancer cells and is controlled by long non-coding RNA NEAT1
Source: Int J Med Sci. 2020 Aug 19;17(14):2214–24. doi: 10.7150/ijms.45706 (PMC7484675; doi:10.7150/ijms.45706)
Supplement: Supplementary file 1 — Supplementary figure S1. [file ijmsv17p2214s1.pdf]

	Predicted consequential pairing of target region (top) and miRNA (bottom)	Site type	Context++ score	Context++ score percentile	Weighted context++ score	Conserved branch length	P_{CT}
Position 1042-1048 of NQO1 3' UTR<br>hsa-miR-129-5p	5' ...AAAAAUAGUCUUCCACAAAAAAU...<br>|||||| 3' CGUUCGGGUCUGGCGUUUUUC	7mer-A1	-0.03	47	0.00	0.635	< 0.1
Position 381-387 of NQO1 3' UTR<br>hsa-miR-211-5p	5' ...UGUAGUUUAUGGCAGAAGGGAAU...<br>|||||| 3' UCCGCUUCCUACUGUUUCCCUU	7mer-A1	-0.08	76	-0.01	3.379	< 0.1
Position 764-770 of NQO1 3' UTR<br>hsa-miR-214-5p	5' ...CCUAGUAGCUGGGAUGACAGGCG...<br>||||||| 3' CGUGUCGUUCACAUCUGUCCGU	7mer-m8	-0.03	61	0.00	0.122	< 0.1
Position 514-520 of NQO1 3' UTR<br>hsa-miR-218-5p	5' ...GUUACUAAUAUGCCUAGCACAAG...<br>|||||| 3' UGUACCAAUCUAGUUCGUGUU	7mer-A1	-0.12	78	-0.02	0.076	< 0.1

Figure S1. Predicted binding sites in NQO1 3'-UTR among the NEAT1 interacting miRNAs. Three NEAT1 interacting miRNAs including miR-129-5p, miR-211-5p, miR-214-5p, or miR-218-5p were selected and the binding sites in NQO1 3'-UTR region were predicted by TargetScan website (http://www.targetscan.org/vert_72/).
